# An *in situ* assessment of local adaptation in a calcifying polychaete from a shallow CO_2_ vent system

**DOI:** 10.1111/eva.12400

**Published:** 2016-07-27

**Authors:** Noelle M. Lucey, Chiara Lombardi, Maurizio Florio, Lucia DeMarchi, Matteo Nannini, Simon Rundle, Maria Cristina Gambi, Piero Calosi

**Affiliations:** ^1^ Department of Earth and Environmental SciencesUniversity of PaviaPaviaItaly; ^2^ Marine Environment Research Centre ENEALa SpeziaItaly; ^3^ Marine Biology and Ecology Research CentrePlymouth UniversityPlymouthUK; ^4^ CNR‐ISMARLa SpeziaItaly; ^5^ Department of BiologyUniversity of AveiroAveiroPortugal; ^6^ Department of BiologyUniversity of PisaPisaItaly; ^7^ Department Integrative Marine EcologyVilla Dohrn‐Benthic Ecology CenterStazione Zoologica “Anton Dohrn”IschiaNapoliItaly; ^8^ Département de Biologie, Chimie et GéographieUniversité du Québec à RimouskiRimouskiQuebecCanada

**Keywords:** evolutionary constraints, *in situ* transplant, Mediterranean Sea, ocean acidification, phenotypic plasticity, Serpulidae

## Abstract

Ocean acidification (OA) is likely to exert selective pressure on natural populations. Our ability to predict which marine species will adapt to OA and what underlies this adaptive potential is of high conservation and resource management priority. Using a naturally low‐pH vent site in the Mediterranean Sea (Castello Aragonese, Ischia) mirroring projected future OA conditions, we carried out a reciprocal transplant experiment to investigate the relative importance of phenotypic plasticity and local adaptation in two populations of the sessile, calcifying polychaete *Simplaria* sp. (Annelida, Serpulidae, Spirorbinae): one residing in low pH and the other from a nearby ambient (i.e. high) pH site. We measured a suite of fitness‐related traits (i.e. survival, reproductive output, maturation, population growth) and tube growth rates in laboratory‐bred F2 generation individuals from both populations reciprocally transplanted back into both ambient and low‐pH *in situ* habitats. Both populations showed lower expression in all traits, but increased tube growth rates, when exposed to low‐pH compared with high‐pH conditions, regardless of their site of origin suggesting that local adaptation to low‐pH conditions has not occurred. We also found comparable levels of plasticity in the two populations investigated, suggesting no influence of long‐term exposure to low pH on the ability of populations to adjust their phenotype. Despite high variation in trait values among sites and the relatively extreme conditions at the low pH site (pH < 7.36), response trends were consistent across traits. Hence, our data suggest that, for *Simplaria* and possibly other calcifiers, neither local adaptations nor sufficient phenotypic plasticity levels appear to suffice in order to compensate for the negative impacts of OA on long‐term survival. Our work also emphasizes the utility of field experiments in natural environments subjected to high level of *p*CO_2_ for elucidating the potential for adaptation to future scenarios of OA.

## Introduction

Ocean acidification (OA) is the process by which anthropogenically derived atmospheric carbon dioxide (CO_2_) is absorbed into surface seawater, lowering the pH and concentration of carbonate ions in the global ocean (Caldeira and Wickett 2003; Doney et al. 2009). These changes have a large potential to impact marine biodiversity, as many marine species are expected to be affected detrimentally (Kroeker et al. 2013; Wittmann and Pörtner 2013; Mostofa et al. 2015). Adaptation may be the most realistic option for survival, but our understanding of the scope for marine species to adapt to ongoing global change over realistic, multidecadal timescales is limited (Hendry and Kinnison 1999; Carroll et al. 2007; Kelly and Hofmann 2012; Sunday et al. 2013). One way to test explicitly if and how species might be able to respond to future oceanic conditions is through studies of local adaptation along natural pH gradients (Bell and Collins 2008; Sanford and Kelly 2011).

The scope for local adaptation in response to environmental stressors has previously been investigated using natural environmental gradients and the responses from their residing populations (Reznick and Ghalambor 2001; Kawecki and Ebert 2004; Gaston et al. 2009; Sanford and Kelly 2011; Dam 2013). These studies have provided us with an understanding on how and why natural populations succeed or fail to adapt to particular stressful conditions and demonstrate realistic ecological scenarios for species’ adaptation to OA (Rodolfo‐Metalpa et al. 2011; Maas et al. 2012; Calosi et al. 2013; Lewis et al. 2013). The majority of examples of contemporary adaptation have shown that new populations are established by colonization events (Reznick and Ghalambor 2001), where a subset of a metapopulation is subjected to a modified environmental patch within the preexisting range of the species (Reznick and Ghalambor 2001). Individuals that initially colonize and proliferate in these new environments can become isolated from their ancestral population and may later radiate and establish a new metapopulation from which distinct populations and species arise through gene flow and repeated colonization [e.g., Losos and Schluter 2000; see also ‘The Rockall Paradox’ Johannesson (1988)]. This type of colonization is likely to be the most effective for the investigation of adaptation to OA, as future chemistry changes will be global, encompassing many species’ preexisting ranges, and initially intensifying current low‐pH areas (i.e. estuaries, fiords, coastal areas, and upwelling areas) (Barton et al. 2012; Hofmann et al. 2014).

There is limited work on local adaptation in response to OA (see Sanford and Kelly 2011). The evidence presented so far indicates that local adaptation to low pH may have occurred in populations inhabiting naturally, low‐pH upwelling areas. For example, populations of the purple sea urchin, *Strongylocentrotus purpuratus*, found in persistent upwelling waters on the west coast of the USA appear to be less sensitive than those that are not exposed to low‐pH waters (Kelly et al. 2013; Pespeni et al. 2013). A similar distinction implying locally adapted populations is further exemplified in studies of highly calcified coccolithophore, *Emiliania huxleyi*, strains that dominate low‐pH upwelling habitats in Chile (Beaufort et al. 2011; Smith et al. 2012). Supporting this idea, laboratory studies have also demonstrated that the *E. huxleyi* upwelling strains have a high degree of adaptation potential compared with strains that are not found in high CO_2_ conditions (Iglesias‐Rodriguez et al. 2008); also see Langer et al. (2009) for counter evidence.

Ocean acidification adaptation studies can also be established in venting areas where volcanic CO_2_ bubbles through the seafloor and locally lowers pH (Hall‐Spencer et al. 2008; Fabricius et al. 2011; Johnson et al. 2013). A vent site off the island of Ischia, Naples, in the south of Italy is one such example. Underwater CO_2_ volcanic emissions interact with a *Posidonia oceanica* sea grass habitat off the coast of the Castello Aragonese peninsula. The CO_2_ bubbles from the sea floor and drives the seawater pH down to equal to – or lower than – business‐as‐usual IPCC projections for 2100 (pH 6.5–7.8; Hartmann et al. 2013), resulting in a low‐pH ecosystem (Hall‐Spencer et al. 2008; Kroeker et al. 2011). As such, the site has been used as an analogue for ecosystems’ responses to the ongoing OA projected to occur in the next century (Hall‐Spencer et al. 2008; Kroeker et al. 2011; Lombardi et al. 2011a).

In most cases, species abundance declines in low pH (Hall‐Spencer et al. 2008; Kroeker et al. 2011); however, a few studies have identified species with higher abundances in low‐pH areas, primarily amphipods and polychaetes (Kroeker et al. 2011; Fabricius et al. 2014; Garrard et al. 2014; Giangrande et al. 2014; Ricevuto et al. 2014; Lucey et al. 2015). This is the case in the low‐pH Castello CO_2_ vent system where several species persist in high abundance and provide an opportunity for testing for the presence of local adaptation (Rodolfo‐Metalpa et al. 2011; Calosi et al. 2013; Lucey et al. 2015).

Reciprocal transplant experiments can be used to determine levels of adaptation among populations living in low‐pH areas and whether the persistence of the population is enabled by forms of adaptation to low pH (Etterson and Shaw 2001; Ayrinhac et al. 2004). In this approach, individuals are taken from different field habitats and held in their respective ‘habitat’ conditions for multiple generations. Following this grow‐out period, their progeny are relocated to the *in situ* source and test habitats, after which their fitness (e.g. survival) is quantified (Falconer and Mackay 1996; Kawecki and Ebert 2004). The performance of local genotypes can then be explored using reaction norms and analysis of variance to test for local adaptation (significant differences between trait means between populations), plasticity (significant effects of environment), or genotype by environment interactions (Nuismer and Gandon 2008). To our knowledge, no studies so far have used the reciprocal transplant approach to test for local adaptation to low‐pH habitats in this context.

Consequently, the aim of our study was to use a reciprocal transplant approach to investigate whether there is evidence for local adaptation and/or plasticity in response to natural exposure to low‐pH conditions representative of future OA in the Castello CO_2_ vents. The study species is a Spirorbinae polychaete (Serpulidae), *Simplaria* sp., which is able to subsist in the naturally low‐pH vent habitat (N. Lucey, C. Lombardi, M. Florio, S. D. Rundle, M. C. Gambi and P. Calosi, unpublished data). The first and only Mediterranean Sea record of this Serpulidae species to date was in the Castello CO_2_ vents during this study. The population in the vents may have relocated to this site sometime during the last 40 years from the Caribbean Sea where it was described as *Pileolaria quasimilitaris* Bailey, 1970 (now *S. quasimilitaris* according to Knight‐Jones 1984). The species could also be a morphotype of *Simplaria pseudomilitaris* (Thiriot‐Quiévreux, 1965) first described in Marseille, France. Alternatively, it may be a new species to science, as the morphological characters of the vent individuals are not in complete agreement with that of either *S. quasimilitaris* or *S. pseudomilitaris*, with distinctive and unique operculum spine morphology (i.e. more abundant, longer, pronounced distally projecting calcareous spines covering the operculum plate (N. Lucey, C. Lombardi, M. Florio, S. D. Rundle, M. C. Gambi and P. Calosi, unpublished data). Generally, Spirorbinae are small, filter feeders that spend their adult lives within self‐built spiraled tubes that are permanently attached to a substrate (Gee 1964; Potswald 1968; Tanur et al. 2010). They are common members of the benthic community, especially in early substrate colonization or as epibionts on other organisms (Rouse and Pleijel 2001). These polychaetes are responsive to rapid evolutionary change through adaptation (Macdonald 2003). A key aspect of their suitability for adaptation studies is their life history: they incubate their embryos in specialized operculum brood chambers (Bailey 1970; Macdonald 2003) and release nonfeeding lecithotrophic larvae that settle quickly, limiting their dispersal (Beckwitt 1981; Kupriyanova et al. 2001). This can result in patchy distributions and significant genetic differences among populations found less than 10 m apart from seemingly identical habitats, with no apparent barriers to mutual colonization (Beckwitt 1981). Additionally, it is thought that these brooders have radiated more rapidly than any other clade in the family (Macdonald 2003). Spirorbinae also serve as an excellent taxon for multigenerational studies as they can be easily cultured under laboratory conditions and have relatively short generation times (~90 days, Kupriyanova et al. 2001).

Previous work on calcifiers in the Castello CO_2_ vents identified *Simplaria* sp. as the dominant and most abundant calcifying polychaete species living in moderately low‐pH (~7.7) areas, with respect to population sizes in ambient seawater sites nearby (N. Lucey, C. Lombardi, M. Florio, S. D. Rundle, M. C. Gambi and P. Calosi, unpublished data). It is also the only species of Spirorbinae polychaete growing on the *P. oceanica* sea grass leaves in an aragonite–calcite tube to maturation within the pH range 6.6–7.7 (N. Lucey, C. Lombardi, M. Florio, S. D. Rundle, M. C. Gambi and P. Calosi, unpublished data). With this study, we tested whether the population of *Simplaria* sp. from the low‐pH site (7.7) would have a significantly greater tolerance to low pH *via* plasticity and/or local adaptation in comparison to that settled in ambient pH (8.1) originating populations. This also allowed us to show how the reciprocal transplant approach can be utilized to find evidence for, or lack of, adaptation to environmental drivers, which may be helpful in informing future conservation and resource management actions within the context of the global change.

## Materials and methods

### Field site and experimental design

**Figure 1:**
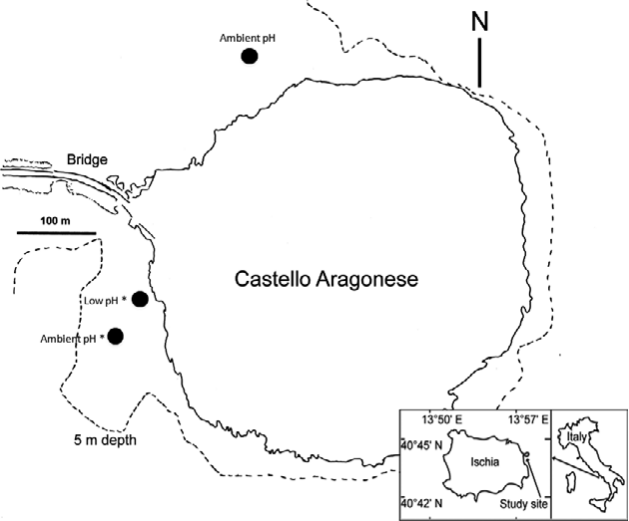
Schematic representation of the study area at the Castello Aragonese off Ischia island (Naples, Italy), showing the sampling locations of both ambient and low‐pH demes (black dots), transplant locations (*) and *Posidonia* meadows (black dashed lines).

Local population samples from within the larger calcifying polychaete *Simplaria* sp. metapopulation living around the Castello CO_2_ vent system were collected from two habitats in the *P. oceanica* sea grass meadow: a low‐pH site (7.69 pH) and two ambient pH sites (8.03 pH). The low‐pH site was selected as the area within the CO_2_ vents where *Simplaria* sp. was found in higher abundance compared with the ambient sites (N. Lucey, C. Lombardi, M. Florio, S. D. Rundle, M. C. Gambi and P. Calosi unpublished data). The ambient sites were located between 100 and 400 m away from the low‐pH site and correspond to the control areas selected for a recent study on the colonization of *Posidonia* sea grass mimics (Donnarumma et al. 2014). Research of closely related spirorbid species has determined their dispersal range is on the scale of a few meters (~10 m), supporting the probability of genetic separation between collection sites in the *Simplaria* sp. in this study (Beckwitt 1981; Kupriyanova et al. 2001). The locations for these sites are shown in Fig. 1.

**Figure 2:**
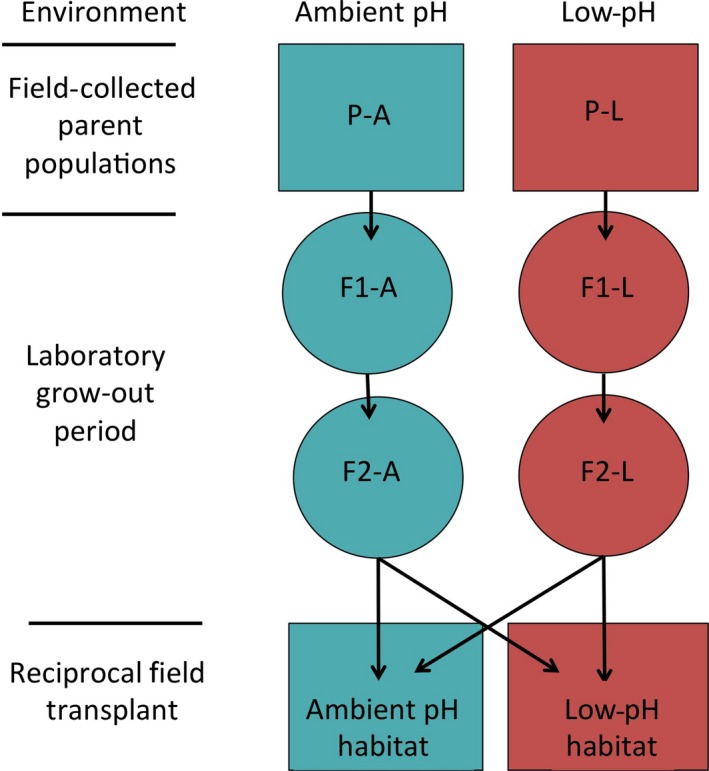
Experimental design schematic illustrating the pH environment of both populations; field stages are represented as boxes and laboratory grow‐out stages as circles. Black arrows indicate reciprocal transplants.

In order to investigate the presence of local adaptation to low‐pH conditions within the Castello CO_2_ vent system, we used a reciprocal transplant experiment to compare the fitness responses of second‐generation offspring raised from grandparents from both pH habitats grown in their source conditions before being reciprocally transplanted into both field habitats (see Fig. 2 for experimental schematic; Kawecki and Ebert 2004). We specifically refer to low and ambient pH populations using the term ‘deme’ to represent the subset of our sampled population (Kawecki and Ebert 2004).

Each starting deme consisted of 500 adult individuals, a quantity regarded as suitable to minimize inbreeding or genetic drift effects in the subsequent generations (Colin and Dam 2005; Dam 2013). Adults collected from the two ambient pH field sites were mixed together to achieve the maximum genetic diversity within the ambient deme and provide comparably sized low and ambient pH starting demes (Nacci et al. 1999). The individuals that were used in the reciprocal transplant experiment were 4‐ to 20‐day‐old F2‐generation recruits bred in laboratory grow‐out conditions resembling the local pH habitats from which their respective field‐collected grandparents were found (Fig. 2). Using F2‐individuals from controlled laboratory conditions minimized any field‐based plasticity and transgenerational effects that may have occurred if field‐collected or F1‐generation individuals were used (in accordance with similar experimental designs; Dam 2013). All F2 recruits transplanted into ambient pH were transplanted to the field site closest to the low‐pH site. Deme‐level replication by pH habitat was not feasible for two reasons. Firstly, we were required to comply with site‐based restrictions using minimal transplants within the sensitive site. Boating traffic related to the summer season causes heavy disturbances in many of the alternative transplant sites. Additionally, a preliminary abundance and distribution survey before the collection of adults for this experiment found that the species was not highly abundant in either of the control or low‐pH areas; therefore, any statistically relevant replication within the site would have jeopardized the *Simplaria* sp. population through oversampling.

The experimental F2 individuals from both demes were left in the field for a 66‐day period before being recovered, preserved and assessed for survival, development, reproductive output, population growth, and tube growth rates. This time frame was chosen to allow adequate time for growth to maturation and was based on life span projections of similar species (Kupriyanova et al. 2001).

### Adult collection

Collection of live adults was performed between the 29th and 30th of September 2014 by SCUBA diving. This involved cutting *Posidonia* leaves with visible tubes from each pH site and placing them in closed fabric bags, keeping the low‐pH and ambient originating individuals separated and in their original seawater pH conditions. The seawater pH at each collection site was taken with an integrated pH meter (SG2; Mettler‐Toledo, Leicester, UK) and refractometer (V2; TMC, São Julião do Tojal, Portugal) (*n* = 3) and was 7.61 ± 0.26 at the low‐pH site and 8.03 ± 0.05 and 8.03 ± 0.08 at both ambient sites. The temperature in the entire area was 21.96 ± 1.29.

**Figure 3:**
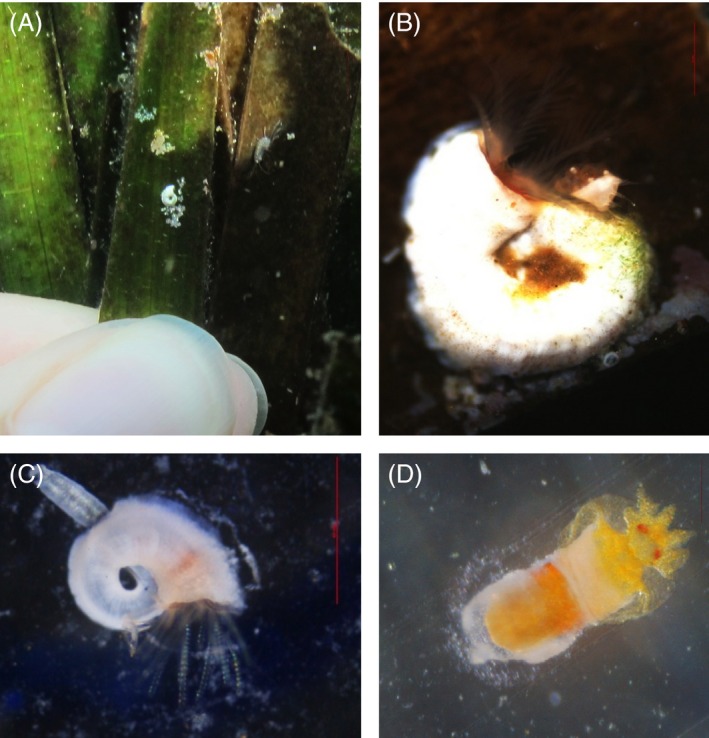
*Simplaria* individuals: (A) adult settled on *Posidonia* blade, scale: 10 mm, (B) close‐up of mature adult with embryos inside the operculum brood chamber, scale: 0.5 mm, (C) juvenile settled on glass settlement slide, scale: 0.5 mm, and (D) postmetamorphic juvenile on glass settlement slide, scale: 0.1 mm.

The material was then transported by boat to the Villa Dohrn‐Benthic Ecology Center of the Stazione Zoologica in Ischia (approx. 4 km from the Castello CO_2_ vent's system; transport time <1 h) and maintained inside 10 L coolers with fresh seawater from each of the collection sites. Once at the laboratory, leaves were immediately trimmed to eliminate as much leaf material surrounding the spirorbid tubes as possible to help in avoiding undesired fermentation. Individual worms were then sorted by their tube spiral direction and operculum morphology, the main taxonomic characters that are considered in living individuals to identify the genus and species (Bailey 1970). All individuals matching the adult *Simplaria* sp. description (N. Lucey, C. Lombardi, M. Florio, S. D. Rundle, M. C. Gambi and P. Calosi, unpublished, Fig. 3) were maintained in 1.3 L aquaria in seawater matching their respective source pH levels.

Individuals were then transported to the Marine Ecology Laboratory of the Marine Environmental and Sustainable Development Unit (ENEA, La Spezia, Italy) by ferry and car on October 3, 2014. During the 8h transport, they were maintained in separate containers with unfiltered seawater (volume: 1.3 L; temperature: 22°C; pH: ambient = 8.15 or low = 7.70; salinity = 36; density: approx. 100 per aquaria). All containers were kept in styrofoam coolers packed with ice to maintain a consistent water temperature. Temperature and pH were recorded twice during the duration of the transport using a pH meter with integrated thermometer (SG2, Mettler‐Toledo). The mean pH in the containers remained at 8.15 (ambient samples), or increased from 7.70 to 8.00 (low‐pH samples), while the temperature decreased from 22 to 19°C for 1 h in all containers during the 8h transportation period.

### Breeding and rearing F2 demes

On arrival at the ENEA laboratory, the specimens from ambient and low‐pH habitats were immediately placed in two experimental seawater culturing aquarium systems simulating their respective pH field habitats within the containers they were transported in (with 100 adults per container). Water parameters in the culturing systems were stabilized within the following 24‐h period to values based on the averages (and standard deviations) of six time series datasets between 2008 and 2015 at each pH habitat, with the low‐pH grow‐out aquarium system set at 7.7 (7.69 ± 0.32), and the ambient at 8.1 (8.03 ± 0.08) (Ricevuto et al. 2014). The temperature was set at a constant 22°C, the average temperature at the Ischia site the previous year during the same time frame of the planned field experiment. Temperatures in this dataset did range from 17.5 to 25.6°C, but we did not account for this variability in the laboratory, as the standard deviation was relatively low (1.23°C).

**Figure 4:**
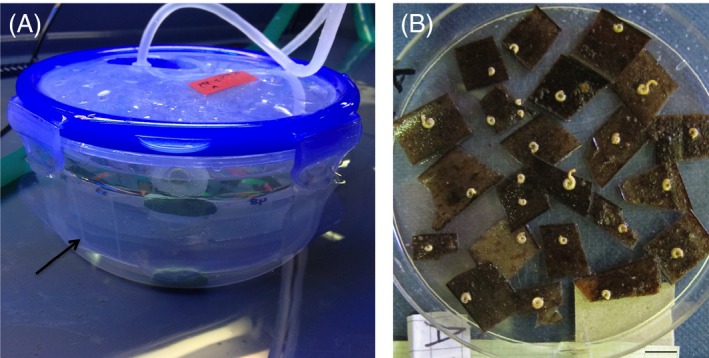
(A) Larval catchment containers lined with glass slides (indicated by black arrow) and (B) field‐collected adult individuals on *Posidonia* leaf sections (grandparents).

After a 2‐day exposure period to laboratory conditions, 16 glass ‘catchment slides’ pretreated with a biofilm from a 24‐h‐filtered seawater soak were positioned along the sides of each container as a substrate for F1 juvenile settlement (Fig. 4). Parents on leaf sections were kept in the containers bordered with catchment slides at a density of 100 individuals per container for one month (Fig. 4). After this period, the containers were disassembled and parents removed. Slides with F1 recruits were placed in slide stands in their respective pH grow‐out tanks in a vertical position. When individuals from the F1 generation of each deme reached maturity, the process for collecting their offspring on slides was repeated in the same way, but with the slides with F1 adults in the containers instead of the original parent individuals on leaf sections. The low‐pH and ambient pH demes and their seawater were never mixed throughout the entire grow‐out period to ensure no interpopulation breeding or genetic mixing *via* spermcasting occurred.

### Laboratory grow‐out system, animal husbandry, and physico‐chemistry

The two seawater culturing aquarium systems were modified and integrated versions of the equilibration flow‐through systems used by Widdicombe et al. (2009) and Melatunan et al. (2011). Each pH treatment level consisted of two header tanks (volume = 90 L each) of seawater, supplied from one sump (22°C) and aerated with either air (*p*CO_2_ ~ 380 μatm, for pH = 8.2), or CO_2_‐enriched air (*p*CO_2_ ~ 1000 μatm, for pH = 7.7). CO_2_ gas was slowly released into a Buchner flask to enable mixing using a CO_2_ regulator (6000 CO_2_, BOC, La Spezia, Italy). *p*CO_2_ in the air supplied to header tanks was measured continuously throughout the grow‐out period with a CO_2_ gas analyzer (Li‐820; Li‐Cor Biosciences, Lincoln, NE, USA) and adjusted manually to the experimental level when necessary.

From each of the four header tanks, seawater was gravity‐fed at a constant rate (100 mL/min) to each of the five larval catchment containers for each deme (transparent sealed 1.3 L containers), which were held within larger holding trays (volume = 150 L) where excess seawater was allowed to flow and the juvenile‐adult settlement slides were set up in slide stands. These slides were distributed evenly throughout the holding trays and mini submersible circulation pumps (HJ‐311; Aquapump, Mondialfauna, Milan, Italy) created a circular flow around the trays to promote filter feeding and gamete circulation. Slides and larval catchment containers were randomly rotated on a weekly basis. One standard florescent white light was positioned above each experimental tank and put on a 14 h: 10 h = L:D schedule to simulate a diurnal cycle. Seawater overflowed from the experimental trays to the respective sumps and was filtered by protein skimmers (V2Skim 600; TMC). Heaters (V2‐Therm 300 W’ TMC) and submersible circulation pumps (HJ‐311; Aquapump) were also used in each sump to maintain stable temperature conditions and enhance a homogeneous mixing of the seawater that was then recirculated *via* a submersible pump (V2 Power Pump 2200; TMC) to the header tanks.

All seawater was collected from La Spezia bay, La Spezia, Italy, approximately every 3 weeks and filtered using a 0.1 μm and UV sterilization filtration system (V2ecton 120; TMC) for 5 days before being introduced to the systems. Partial water exchanges were made with this filtered seawater, exchanging approximately 400 L per system every 2 weeks. Additionally, a food mixture of rotifers, *Artemia*, and microalgae for filter feeders was added to each system at a concentration of 3 mL feed per 300 L of seawater twice weekly (Gamma Nutraplus Reef Feed; TMC). On feeding days, the food mixture was injected near the growing individuals and protein skimmers were turned off for a 24‐h period to provide adequate time for *ad lib*. feeding. These laboratory‐based feeding conditions were chosen to best match the food composition and availability found in the natural *Posidonia* habitats in both pH sites (Ricevuto et al. 2014), preventing any growth restrictions due to diet. Deionized water was added daily to keep the salinity stable around 38 ± 1.

During the laboratory grow‐out period, the seawater pH, temperature, and salinity were measured daily in each larval catchment container and holding tray with an integrated pH meter (SG2; Mettler‐Toledo) and refractometer (V2; TMC) (*n* = 6). The pH meter was calibrated daily with pH buffer standards (4.01, 7.0, 9.21; Mettler‐Toledo). The pH in the low and ambient conditions averaged 7.79 ± 0.10 and 8.22 ± 0.07, with temperatures of 22.38 ± 1.08 and 22.06 ± 0.96°C, respectively, throughout the 7‐month grow‐out period (Table 1). Seawater samples (250 mL) were taken monthly from the same holding tray locations for total alkalinity analysis (*n* = 3). Samples were fixed with HgCl_2_ (0.02%), stored in borosilicate flasks (250 mL), and maintained in dark, dry conditions until total alkalinity (*A*_T_) was determined using Gran titration method (Dickson et al. 2007). Carbonate system parameters were calculated from *A*_T_, pH, temperature, and salinity using the package SeaCarb v.2.4.8 in software R (Lavigne and Gattuso 2013; Table 1).

**Table 1:** Seawater physico‐chemistry parameters (mean + SD) measured (in bold) or calculated (plain text, determined using the SeaCarb program^a^) during the laboratory grow‐out phase and reciprocal transplant experiment in each pH habitat

	Ambient pH	Low pH
Laboratory: acclimation and grow‐out phase		
**pH** ^d^	8.22 ± 0.07	7.79 ± 0.10
**Temperature** (°C)^d^	22.06 ± 0.96	22.38 ± 1.08
**Salinity** ^d^	38.70 ± 0.99	38.57 ± 0.92
**TA** (μmol/kg)^m^	2469.78 ± 110.15	2434.51 ± 156.66
[CO_2_] (μmol/kg)	732.92 ± 142.69	2545.18 ± 590.09
*p*CO_2_ (ppm)	244.05 ± 43.54	852.65 ± 197.83
[${\text{HCO}}_{3}^{-}$] (μmol/kg)	1704.45 ± 107.19	2097.39 ± 173.84
[${\text{CO}}_{3}^{2-}$] (μmol/kg)	307.99 ± 36.98	135.79 ± 17.88
DIC (mol/kg)	0.002 ± 1.02 × 10^−4^	0.002 ± 1.72 × 10^−4^
Ω calcite	4.70 ± 0.54	2.07 ± 0.27
Ω aragonite	7.16 ± 0.82	3.16 ± 0.40
Field: reciprocal transplant experiment^b^		
**pH** ^h^	8.05 ± 0.05	7.36 ± 0.35
**Temperature** (°C)^h^	24.01 ± 0.51	24.01 ± 0.51
**Salinity** ^m^	37.41 ± 1.34	37.41 ± 1.34
**TA** (μmol/kg)^a^	2401.52 ± 91.70	2283.72 ± 222.54
[CO_2_] (μmol/kg)	1183.30 ± 183.66	3132.11 ± 1484.10^c^
*p*CO_2_ (ppm)	402.82 ± 61.74	5267.93 ± 7332.39
[${\text{HCO}}_{3}^{-}$] (μmol/kg)	1848.59 ± 106.07	2101.41 ± 272.14
[${\text{CO}}_{3}^{2-}$] (μmol/kg)	222.76 ± 22.09	73.02 ± 55.99
DIC (mol/kg)	0.002 ± 9.98 × 10^−5^	0.002 ± 3.59 × 10^−4^
Ω calcite	3.44 ± 0.30	1.13 ± 0.87
Ω aragonite	5.24 ± 0.46	1.73 ± 1.33

Sampling frequency is denoted superscripts, where ‘h’: hourly, ‘d’: daily, and ‘m’: monthly.

a Lavigne and Gattuso (2013).

b Low pH and temperature field site monitoring spanned from June 17 to July 2; ambient pH site data taken from Donnarumma et al. (2014) during similar time periods. Field‐based TA measurements also include data from collection period and time series data from Ricevuto et al. (2014).

c Outlier removed.

### Reciprocal transplant experiment setup

In preparation for the field transplant, slides with postmetamorphic individuals from the F2 generation between age 4 and 20 days from both low‐pH and ambient demes were collected from the laboratory grow‐out systems and photographed with a digital camera (Nikon Sight DS‐U1; Nikon, Milan, Italy) mounted on a light microscope (AZ100; Nikon). Photographs were analyzed with ImageJ software (Rasband WS, US National Institutes of Health, Bethesda, MD, USA) to obtain tube surface area (mm^2^; Abràmoff et al. 2004).

**Figure 5:**
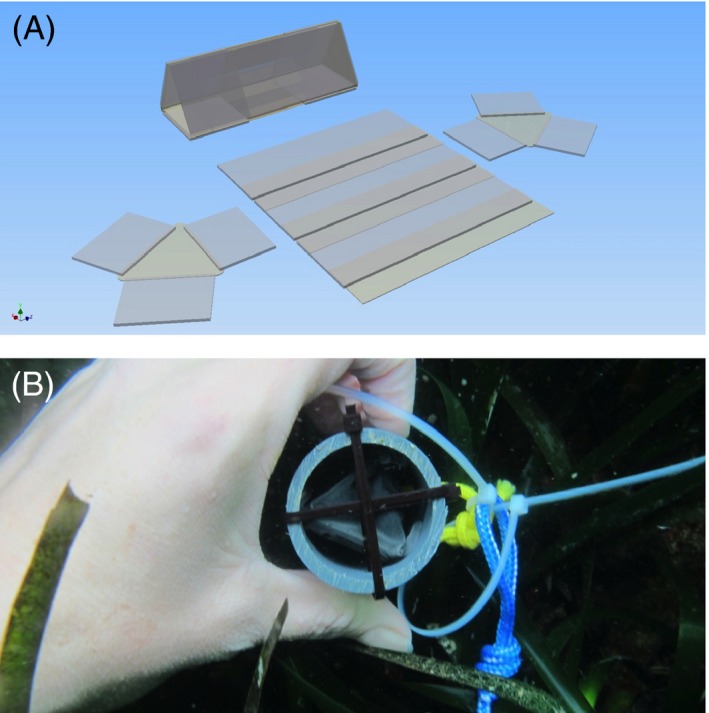
(A) Settlement traps holding laboratory‐grown F2 individuals on glass slides (drawing by J. Paulus) and (B) PVC tubes with animals secured inside the glass triangular prism, capped with mesh and secured to the top of stakes vertically positioned on the seafloor in the *Posidonia* meadow at the sites of both population's origin.

F1 individuals were removed from these slides, as well as any individuals on the back of the slides. This ensured that there was only one slide face with F2 individuals. Each slide had between 1 and 14 individuals. As it was not possible to control how may larvae settled on the slides, we accounted for the resulting variability by placing slides with both low and high numbers of settlers in traps in each treatment. Furthermore, we attempted to balance the number of individuals per treatment by equally dividing the slides between treatment and field stakes. Details of replication levels are given in Table 2. The slides were then inserted into settlement traps that consisted of three faces folded together as a triangular prism with 0.45 μm mesh caps secured on both ends. This mesh size was primarily used to attain F3 data by retaining any trochophore larvae within the trap, as the larvae are approximately 50 μm in diameter (Lucey, per*s. obs*.). The mesh served three additional purposes: (i) preventing any tube loss due to detachment inside the trap, (ii) decreasing the predatory risk from larger crustaceans or fish, and (iii) restricting other polychaete larvae from setting inside traps. Laboratory flow‐through tests using colored dye were performed to determine whether water circulation through the traps differed depending on the presence of mesh caps (i.e. with or without mesh caps). No differences were observed so we assumed that food availability and water parameters inside the traps would be comparable to the field measurements. This glass structure was then was inserted into a PVC tube and secured with thin plastic zip‐ties (Fig. 5).

**Table 2:** Quantity of total individuals in each reciprocal transplant treatment and the number of corresponding traps and stakes per treatment

Treatments	Low pH → low pH	Low pH → ambient	Ambient → ambient	Ambient → low pH
Individuals (#)	25	12	16	33
Traps per treatment (#)	7	6	6	10
Stakes per treatment (#)	5	3	3	5

These field traps were transported to the Benthic Ecology Center of Ischia on April 26, 2015. During the 8h transport, the individuals were maintained in separate containers with filtered seawater (volume = 1.3 L; temperature = 22 ± 3°C; pH: ambient = 8.15 ± 1, low = 7.7 ± 1; salinity = 36; density: 8 traps per container). All containers were kept in styrofoam coolers packed with ice to maintain a consistent water temperature (22 ± 3°C). Upon arrival, traps were put in flowing ambient seawater for one night. Before deployment, mesh caps were visually inspected for detached tubes. Then, three to four traps were attached to rope between 5 and 10 cm long and secured to the top a 40‐cm‐long iron stakes and transferred by boat in coolers with seawater to the field sites, and brought to the seafloor *via* SCUBA diving. Stakes were pushed into the sea grass mat and were vertically positioned on the seafloor in the *Posidonia* meadow at the origin sites of both populations. Attached traps drifted within the sea grass meadow. Ten stakes were prepared for the ambient and low‐pH deme traps, with 10 traps for each of four treatments: (i) Amb‐Amb, (ii) Low‐Low, (iii) Amb‐Low, and (iv) Low‐Amb, with ‘Amb’ as ambient, control pH conditions (8.1), and ‘Low’ as Low pH (7.7). This experimental time frame allowed the F2 individuals to grow into adulthood, and any resulting offspring to settle on the three glass slide surfaces of the trap. The stakes are considered replicates of each treatment nested inside the traps.

### Reciprocal transplant experiment collection and characterization of fitness metrics

The stakes with the traps were collected from the field *via* SCUBA after the 66 days of *in situ* exposure (on July 2). The traps were immediately put into a magnesium chloride solution (75 g/L seawater) to relax the specimens for 20 min, after which they were transferred to 4% neutralized formalin for 24 h. Traps were then immersed in freshwater to rinse formalin out, and immediately transferred into 70% EtOH for long‐term preservation. This was done to prevent formalin, although neutralized, from possibly corroding the calcium carbonate tubes of the worms.

The traps were disassembled, and the contents were examined to find and identify larvae or detached tubes. Recorded positions of individuals on the slides at the start of the experiment were compared to those at the end of the experiment to identify original F2 individuals. Survival at time of collection was defined by determining the presence of worm bodies within the F2 tubes, seen through the underside of the slides: The presence of worm body indicated that the individual was alive throughout the experiment. Photographs of ‘live’ individual tubes were taken and tube surface area measured using ImageJ software described above. Tube growth rates were calculated as the ratio between final tube surface area over initial tube surface area (mm^2^), which also accounted for tube dissolution. Mortality was recorded for any empty tubes or detached tubes found in the mesh without bodies.

To determine the developmental stage of the individuals, tubes were carefully broken open and worm bodies extracted. Individuals were categorized as juveniles, or mature adults with or without embryos. For mature adults with embryos, the operculum was dissected and embryos counted. Reproductive output was measured as the number of additional tubes found inside the trap on any of the three slides plus any embryos found in the F2 operculum brood chamber. When more than one mature F2 adult was present in a trap with F3 recruits, the number of F3 individuals was divided by the number of surviving mature F2 adults in the trap. When more than one F2 individuals was found dead in a trap with living F3 individuals, offspring were assumed to originate from only one F2 parent. F3 growth was defined as the average tube area of all F3 tubes from one adult, determined by photographing new F3 tubes with ImageJ software as described above. F3 individuals with full spirals were dissected to determine developmental stage.

### Reciprocal transplant experiment seawater physico‐chemistry

**Figure 6:**
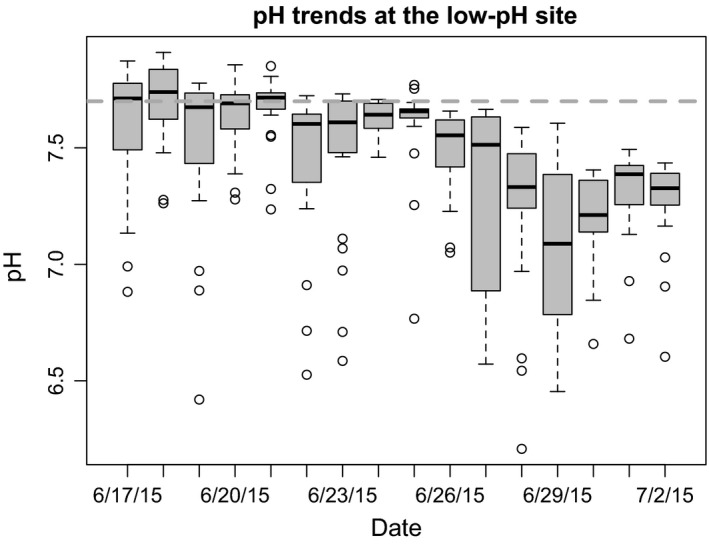
Boxplots representing the daily median, spread, and skewness of pH measurements throughout each day during the reciprocal transplant experiment at the low‐pH transplant site, with the dashed horizontal line depicting the expected average pH (7.7). Measurements taken hourly by a Honeywell Seafet pH sensor stationed approximately 2–4 m from the transplants on the seafloor.

During the field transplant experiment, hourly measurements of pH and temperature at the low‐pH site were recorded with the pH meter (Honeywell Seafet pH sensor; Martz et al. 2010), which was deployed next to the experimental transplant from June 17, 2015, to July 2, 2015 (Table 1, Fig. 6). pH values for the ambient site were taken from past datasets (Table 1). Discreet field measurements of pH, salinity, temperature, and total alkalinity were taken at the field sites at the time of specimen collection and at the experimental end with an integrated pH meter (SG2; Mettler‐Toledo) and refractometer (V2; TMC) (*n* = 3). For total alkalinity, seawater samples (250 mL) were collected from each site (*n* = 3), and sampled with the same methodology used for the laboratory seawater chemistry described above.

### Data analysis

To test the relative importance of ‘deme’ (i.e. potentially different genotypes), ‘habitat’ (i.e. different pH conditions) and their interaction on: (i) F2 survival, (ii) maturation, (iii) reproductive output, as the number of F3 recruits and embryos per F2 parent, (iv) total population growth, as the total number of F2 survivors, embryos and F3 recruits; and (v) F2 tube growth rate, we constructed generalized linear models (GLMs), setting ‘deme’ and ‘habitat’ as fixed factors. Initial ‘tube area’ was set as the covariate, to account for differences in starting size and/or age. Initially, models included ‘trap’ set as a random factor nested in ‘stake,’ which was also set as a random factor nested in ‘habitat.’ As the factors ‘stake’ and ‘trap’ did not exert a significant effect on the study variables, they were removed from subsequent models (Crawley 2012). Interactions and the covariate were retained in all cases. For the traits (i) survival and (ii) development to maturation, we used GLMs with binomial errors. Preliminary data analysis (Zuur et al. 2010) indicated over‐dispersion for the traits (iii) reproductive output and (iv) total population growth, which we corrected using a Poisson GLM, and also corrected the standard errors using a quasi‐GLM model.

Two replicate stakes placed in the ambient habitat went missing, likely due to accidental boat anchor removal, leaving the experimental design with three stakes in the ambient field habitat, and five in the low‐pH field habitat. Regardless, the experimental design included four treatments with a minimum of three stake replicates, and between 12 and 33 individual worm replicates per treatment (see Table 2), and the models employed accounted for this heteroscedasticity (Sokal and Rohlf 1995). Additionally, in each final model, nonsignificant terms are retained to show all interactive effects. To further validate the final models, nonsignificant terms were sequentially dropped until the minimal adequate model was reached (Crawley 2012), and the fit of these simplified models was assessed by plotting residuals against fitted values to check for mean residual deviation of zero and constant variance.

Differences between tube areas from F3 recruits in the ambient habitats from both demes were analyzed using a one‐way anova. No interaction could be tested with F3 tube area, as there was an insufficient quantity of individuals from the low‐pH habitat.

All statistical analyses were performed using the statistical software R v.3.1.3 (R Core Team 2015).

## Results

### Pre‐experiment grow‐out recruitment and survival

The first F1 recruit from the ambient deme was observed 5 days after relocation to the laboratory, whereas the first low‐pH deme F1 recruit was observed 7 days after relocation (Table 3). Field‐collected parents continuously reproduced during the first month, which resulted in 599 F1 recruits from the ambient and 1076 F1 recruits from the low‐pH demes. Greatest mortality levels were observed within the first 3 months of recruitment with 76% mortality in the low‐pH and 55% in the ambient deme. In the following 4 months, mortality was observed to decrease to 45% and 24%, in the low‐pH and ambient pH demes, respectively. The low‐pH deme's mortality was comparably higher throughout the grow‐out period; however, mortality levels for both ambient and low demes were equal at the 4‐month mark (Table 3).

**Table 3:** Laboratory deme population sizes and maturation times for both F1 and F2 during the laboratory grow‐out phase

	Low‐pH deme	Ambient pH deme
Initial parent population size: Oct. 4, 2014	500	500
F1 Population size at maturation (date of first recruit)	143 (Oct. 10)	204 (Oct. 8)
F2 Population size at transplant (date of first recruit)	67 (April 7)	164 (March 6)

Approximately 35% of the initial F1 recruits from the ambient deme survived and became reproductively mature adults. In the low‐pH deme, only 13% of the initial F1 recruits survived to maturity, although the actual quantities of mature individuals were not as dissimilar, with respect to initial recruitment, with quantities of 143 mature individuals in the low pH and 204 in the ambient pH. Additionally, the time for the first F1 individual to reach maturation was 5 months in ambient deme versus 6 months in low‐pH deme (Table 3). At the time of field transplant (April 27, 2015), F2‐deme sizes reflected the disparity in F1 developmental timing, as the ambient F1 deme had an additional 30 days to produce F2 offspring, compared with the low‐pH deme, and was 40.9% larger in overall size (164 individuals vs 67).

### Transplant experiment

**Figure 7:**
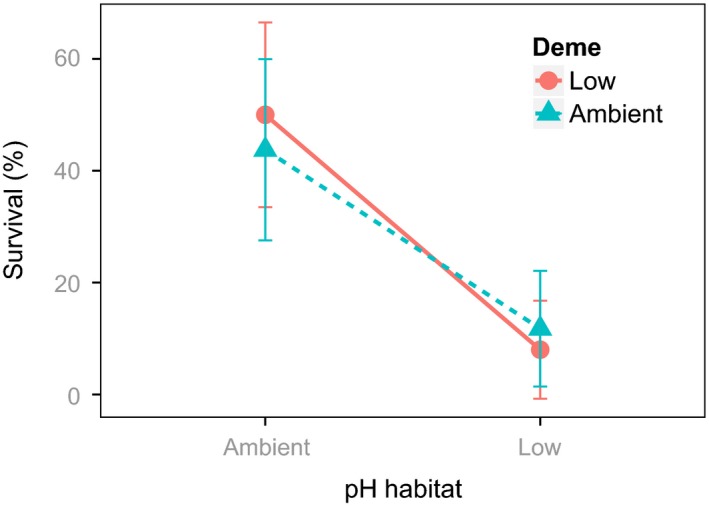
Reaction norm of percent survivorship in second‐generation (F2) *Simplaria* sp. individuals from both the low‐pH deme (red solid line) and ambient pH deme (blue dotted line), transplanted into both pH habitats (8.1 and 7.7). Points are mean ± SE.

**Figure 8:**
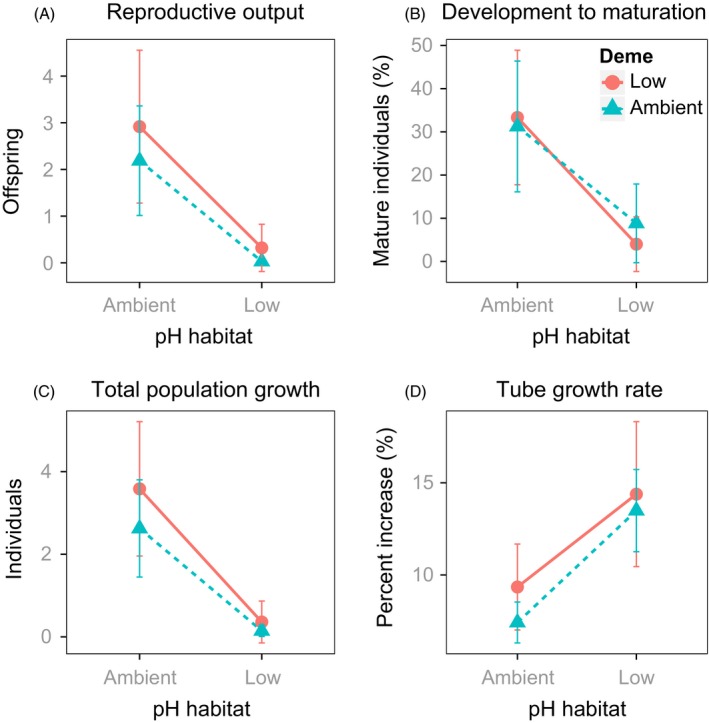
Reaction norms for fitness‐related traits assessed in second‐generation (F2) *Simplaria* sp. individuals from both the low‐pH deme (red solid line) and ambient pH deme (blue dotted line), transplanted into both pH habitats (8.1 and 7.7): (A) reproductive output from all individuals, (B) percent of individuals developing to maturity in the field, and (C) total population size, as living individuals plus their embryos and settled juveniles of individuals, and (D) percent increase in tube growth rates. Points are mean ± SE.

We observed comparable, significant reductions in the survival, development to maturity and total population growth in both *Simplaria* sp. demes transplanted to the low‐pH habitat, and found no significant effect of ‘deme’ or the interaction between ‘deme’ and ‘habitat’ (Figs 7 and 8, Table 4). There was also a reduction in the number of F3 embryos and recruits produced (reproductive output) following exposure to the low‐pH habitat in both demes, yet this decline was only marginally significant (Fig. 8, Table 4). The low‐pH deme was, however, able to reproduce more than once during the field period, whereas the ambient pH deme did not. For both demes, tube growth rates were twice as high when they were exposed to the low‐pH habitat – a difference that was significant (Fig. 8, Table 4). There was no significant difference between demes, however, and reaction norms were the same for all traits (Figs 4 and 8). In each of the traits described above except for survival, the initial tube area was a significant covariate, with low‐pH demes having smaller tubes, compared with ambient pH demes. This was likely negative for every trait except tube growth rates.

**Table 4:** Results of GLMs investigating the effect of deme (genotype = G) and habitat (environment = E) on survival, maturation, reproductive output, total population growth, and tube growth rate in the calcifying spirorbid *Simplaria* sp. (with initial tube area as a covariate)

Trait	Estimate	SE	*Z* value	*P* value
Survival				
Intercept	−1.495	0.797	−1.876	0.061
Deme (G)	0.647	0.804	0.806	0.421
Habitat (E)	−2.083	0.822	−2.534	**0.011**
Interaction (G × E)	−0.383	1.245	−0.308	0.758
Initial tube area (cov)	10.826	5.669	1.910	0.056
Maturation				
Intercept	−2.704	1.002	−2.699	**0.007**
Deme (G)	0.702	0.889	0.790	0.430
Habitat (E)	−2.111	1.001	−2.109	**0.035**
Interaction (G × E)	−0.400	1.557	−0.257	0.797
Initial tube area (cov)	16.322	7.567	2.157	**0.031**
Reproductive output				
Intercept	−0.341	0.666	−0.513	0.610
Deme (G)	0.856	0.639	1.340	0.184
Habitat (E)	−4.233	2.286	−1.852	0.068
Interaction (G × E)	2.030	2.450	0.828	0.410
Initial tube area (cov)	6.867	2.421	2.836	**0.006**
Population growth				
Intercept	−0.052	0.540	−0.096	0.924
Deme (G)	0.813	0.524	1.553	0.124
Habitat (E)	−2.823	0.986	−2.864	**0.005**
Interaction (G × E)	0.530	1.246	0.426	0.671
Initial tube area (cov)	6.378	2.022	3.154	**0.002**
F2 tube growth rate				
Intercept	2.415	0.184	13.110	**<0.005**
Deme (G)	0.077	0.205	0.376	0.707
Habitat (E)	0.676	0.195	3.462	**<0.005**
Interaction (G × E)	−0.270	0.308	−0.876	0.381
Initial tube area (cov)	−2.996	0.988	−3.033	**0.002**

Bold values indicate a statistically significant effect (*P* < 0.05).

Additionally, the F3 recruits grew to similar sizes in the ambient habitat regardless of parent deme identity, with F3 recruits from low‐pH demes having a mean tube surface area of 0.33 ± 0.06 mm^2^, and recruits from ambient pH demes having a mean tube surface area of 0.28 ± 0.06 mm^2^ (*F*_1,7_ = 0.381, *P = *0.539). In both low‐pH treatments, only one F3 tube was found, indicating that low pH severely inhibits tube production regardless of deme identity. This F3 tube was from the low‐pH deme.

## Discussion

Our reciprocal transplant experiment provides no evidence that local adaptation in the *Simplaria* sp. population living under low‐pH conditions has occurred. Furthermore, we show that worms’ phenotypic plastic responses could not compensate for the negative effects of exposure to low pH by improving fitness. This suggests that multigenerational exposure to low‐pH conditions within the CO_2_ vents has not imposed selection for *Simplaria* sp. genotypes that are tolerant to extreme pH variability and low pH (~7.36). Moreover, the conditions at the low‐pH vent site seem responsible for reducing the mean fitness of individuals originating from both low and ambient pH habitats. These results stand in contrast to previous research on polychaetes and other marine calcifiers showing extensive, rapidly evolving adaptive divergence when exposed to pollution, elevated temperature, changes in *p*CO_2_, and even multistressors (Grassle and Grassle 1977; McMullin et al. 2000; Lohbeck et al. 2012; Pansch et al. 2014; Schluter et al. 2014; Parker et al. 2015; Rodríguez‐Romero et al. 2015; Thor and Dupont 2015). Below we discuss the possible constraints to local adaptation in this low‐pH habitat and emphasize the complexities involved in predicting evolutionary patterns, as well as the need for further work with more replication to further validate these findings (Bell and Collins 2008). We then consider how the *Simplaria* sp. response might reflect the inherent inability of certain species to adapt to the stressful OA conditions expected to occur (Dupont and Pörtner 2013).

### Local adaptation constraints

Many studies have indicated that phenotypic plasticity might enhance the process of adaptation by moving the phenotype closer to the fitness optimum for genetic selection, for example, adaptive plasticity (Pigliucci 2005; Ghalambor et al. 2007). Here, the responses do not seem to strongly support previous findings where plasticity was considered to be a precursor to adaptation (Merilä and Sheldon 2000; Réale et al. 2003; Rodríguez‐Romero et al. 2015). Neither of the *Simplaria* sp. demes investigated here have different plastic responses in any of the fitness traits, indicating an inability to change fitness outcomes through plasticity. However, the general depressed fitness in low pH of both demes compared with those in ambient pH habitats indicates a possible nonadaptive plastic response at the metapopulation scale (Huey and Berrigan 1996; Calosi et al. 2013; Turner et al. 2015). Nonadaptive plasticity is where phenotypic changes do not directly contribute to increased fitness under the changed conditions, and it has primarily been associated with limiting population persistence (Chevin et al. 2010). However, current work has recently revived the idea that nonadaptive plasticity may also facilitate adaptive genetic changes by increasing the strength of natural selection (Ghalambor et al. 2015; Merila 2015).

In contrast to the uncertain role of plasticity, bottleneck and genetic drift effects are mechanisms thought to hinder adaptation and speciation (Coyne and Orr 2004). The brooding nature of the *Simplaria* sp. could naturally result in genetic drift effects compared with species with greater dispersal potential. However, genetic drift may eventually result in adaptation (Gavrilets and Hastings 1996), as demonstrated by the numerous brooding species found with local adaptations (Sanford and Kelly 2011). While no adaptation was evident at the time of this study, expanded distribution surveys of *Simplaria* sp. and experimental replication at alternate vent sites, as well as along varying temporal periods in the same site, would help to determine whether genetic drift is affecting this population, and if improved fitness through genetic drift is possible.

Increased magnitude and frequency of the selective driver through time, in this case pH, is likely the most pertinent explanation for the lack of observable genotype environment interactions, and the significant effect of low‐pH ‘habitat’ on reduced fitness (Hoffmann and Sgrò 2011). The mean pH in the low‐pH habitat during the transplant was on average 0.43 pH units lower than the low pH rearing conditions in the laboratory (*in situ* pH range: 6.14–7.90; mean ± SD: 7.36 ± 0.35, *p*CO_2_ > 5000 μatm, Table 1). Increased CO_2_ venting was likely the general cause for the observed low pH, as no pH measurements during the last week of the experiment were at or above the expected pH (Table 1, Fig. 6). Additionally, hourly measurements in the field showed consistent patterns of severe pH fluctuation, where pH decreased each night before increasing the next day (Fig. 6, outliers), the latter likely being the result of sea grass and algae diurnal photosynthetic activity. The field site's pH intensity and frequency (i.e. variability) was not replicated in the laboratory, nor was it expected during the experiment. During the past 6 years of pH monitoring at the low‐pH site, the pH was highly variable (Ricevuto et al. 2014). However, pH only reached levels lower than those recorded here in one study (Calosi et al. 2013; pH 7.19), and only for one documented week. While grandparents of the low‐pH originating deme were subjected to previous natural pH fluctuations, the levels of the pH during the experiment may have surpassed a pH threshold (Scheffer et al. 2001; Dupont et al. 2009; Christen et al. 2013), resulting in the overall lowered fitness of both *Simplaria* sp. populations in the low‐pH site.

The sea grass may also be creating a microenvironment that adds to the magnitude of the low‐pH fluctuations, potentially causing effects to the spirorbids settled on it (Garrard et al. 2014; Wahl et al. 2016). High levels of photosynthesis are thought to provide a refuge from low‐pH conditions during the day; sea grass can create a localized change in pH up to 1 unit higher according to Hendriks et al. (2014). An example of this effect was observed in the spirorbid *Spirorbis spirorbis* settled on the algae *Fucus serratus* (Saderne and Wahl 2013). In more detail, when the spirorbids were exposed to high *p*CO_2_, a reduction in the growth rate was observed, whereas the calcification response measured during irradiation hours was 40% higher with respect to that recorded during dark hours. Spirorbid presence in low‐pH vent site could therefore be attributable to a pH buffering effect from photosynthetic and respiratory processes of the host sea grass on the carbonate system, if there is a positive net effect throughout the diurnal cycle (Saderne and Wahl 2013). This natural pH variability has not been investigated in the *Posidonia* meadows at a microscale <1 mm, but the dial fluctuations measured in the low‐pH site support the hypothesis that the spirorbids are naturally subjected to high variability due to photosynthetic processes. This type of variability is thought to drive selection to favor high phenotypic plasticity and/or select for more robust genotypes, as seen in Pansch et al. (2014) where barnacle tolerance to naturally low pH was higher when populations originated from highly variable pH environments, compared with nonfluctuating environments. It is therefore possible that the pH variability in the low‐pH site may similarly promote plasticity or robust genotypes during ‘normal’ venting periods, and that high venting periods may degrade any previously developed tolerances (Pansch et al. 2014). The implementation of detailed monitoring of abiotic parameters to future natural evolution experiments will help to resolve the uncertainty regarding how varying scales of temporal pH fluctuations common to coastal systems will influence plasticity and adaptation, an important and overlooked component of constraining evolutionary predictions in the context of global change (Wahl et al. 2016).

Natural pathogens may also have influenced our results (Kawecki and Ebert 2004). Predation and pathogens in populations subjected to environmental change tend to act continuously on the average phenotype, reducing the mean fitness and impeding diversification by reducing population sizes (Van Valen 1973; Morgan and Buckling 2004; Meyer and Kassen 2007; Bell and Collins 2008). In this experiment, we controlled for main predators in the field, such as fish and crustaceans through trap protection and the use of mesh caps on these traps. However, pathogens could not be accounted for as easily. In particular, protozoans appeared to be relatively abundant in low‐pH conditions, but not in ambient pH conditions in the laboratory culture. There is a substantial research body documenting the presence of protozoa on and within spirorbid tubes (reviewed in Kupriyanova et al. 2001), however whether the nature of this relationship causes harm remains inconclusive (Knight‐Jones et al. 1975). Generally, protozoa are associated with consuming bacteria (Barker and Brown 1994); therefore, the increase in protozoa may indicate a change in bacterial communities with low pH (Lidbury et al. 2012). Accounts of bacterial/ microbial communities in low‐pH environments from other venting sites have demonstrated decreased low‐pH tolerance of certain species with changed microbial communities (Morrow et al. 2015). This highlights the importance of indirect community and host interactions in response to low pH, and the need for experiments specifically testing hypotheses regarding multispecies adaptive interactions (see Morrow et al. 2015).

One of the main difficulties of natural evolution studies using small CO_2_ vents is the unattainability of population‐level replication for many species among different vents. This is partly because the populations living in most CO_2_ vent systems are not easily comparable, that is, different ‘tolerant’ populations and/or species (see Kroeker et al. 2011; Fabricius et al. 2014, and differences between the Castello and Papua New Guinea CO_2_ vent dominating species). Many of these systems are also in different regions with different conditions and stressors, for example, Italy, Mexico, and Japan (Boatta et al. 2013; Crook et al. 2016). The small spatial scale of these CO_2_ vent sites also limits the possibility to replicate populations of adequate sizes in order to acquire F2 individuals, as in the Castello CO_2_ vents where population replication was not possible due to the limited quantity of individuals. Due to these caveats, the variability of the results of nonreplicated studies must be cautiously assessed. In our results, high variance in the standard error around the means for each population in most assessed traits occurred (Figs 7 and 8). Despite this, the effect of ‘pH habitat’ was consistently significant among all traits and the interactive effects in all traits were nowhere near significant, suggesting that our findings are reliable. The variability, therefore, likely reflects either the relatively high plasticity of the studied populations, or variability in the small‐scale local conditions that occurred during experimental field exposure. Replicated studies through time and the use of multiple (separated) reference sites might help to overcome these within‐site replication limitations, and also help to determine whether changes in interpopulation variability would result in significant population (deme) effects.

Regardless of the considerations discussed above, our results may be an indication of the inability of the *Simplaria* sp. metapopulation within this system to adapt to highly variable low‐pH conditions. Increased mortality in this species was coupled with decreased reproductive effort and increased growth rates as a result of low‐pH exposure. Increased mortality is not necessarily detrimental to a population if it leads to selection for adaptive changes in life history traits, such as increased reproductive output (see Stearns 1983; Reznick et al. 1990). However, the high mortality in all demes in the low‐pH *in situ* habitat are associated with lower reproductive capacity, leading to lower mean fitness and therefore a reduced opportunity for adaptation (Bell and Collins 2008).

In contrast, increased reproduction and increased mortality in the low‐pH deme was indicative of a trade‐off during the laboratory grow‐out period, where mortality and recruitment in the low pH was twice that of the ambient pH after F1‐generation recruitment. Furthermore, the only individuals able to produce two broods during the field experiment were from the low‐pH deme, despite suffering from high mortality levels in low pH. These high mortality levels alongside increased recruitment levels within the low pH rearing phase align well with the idea that the two populations tested here have two different morphologies (phenotypes). This appears to be a trade‐off that could lead to an adaptive phenotypic response within the population if allowed more time, but also resembles a high risk (Stearns 1989). An alternative explanation for the grow‐out period mortality results may be attributed to natural mortality variability. Comparisons of postsettlement mortality levels ranged from 79.5% to over 90% in populations of similar species of brooding spirorbids (Kupriyanova et al. 2001), indicating that the variance between the mortality levels *Simplaria* populations may be within normal ranges. Further work is necessary to establish this species’ natural mortality levels by comparing the responses of other nearby *Simplaria* sp. populations. Replicated transplant experiments through time could also help to determine whether increased reproduction is a significant adaptive trait or trade‐off (Kawecki and Ebert 2004).

Interestingly, exposure to low‐pH conditions prompted increased tube growth rates in all surviving individuals, which was coupled with decreased maturation and lower reproductive output. Tube growth rate was the only trait that increased in low pH, and notably the only trait that was not directly representative of Darwinian fitness. It is thought that tube size is generally correlated with body size, maturation, and reproductive output in spirorbids (Kupriyanova et al. 2001), but our findings indicate that there may be a nonadaptive trade‐off caused by low‐pH exposure hampering this relationship. For example, the energetically costly activity of mineralization could be detracting resources/energy away from reproductive efforts under low pH (Knoll 2003). In a physiological context, these findings support those of Lombardi et al. (2011a,b), where bryozoans transplanted into low‐pH vent sites switched resource allocation away from defense to favoring rapid growth. Wood et al. (2008) also showed increases in calcification rates and metabolism in a brittlestar, but at the cost of muscle wastage, which was thought to be unsustainable. Furthermore, similar patterns were also found in four other marine calcifiers, where exposure to low pH implied a shift in the energy budget expenditure away from survival‐related processes and into calcification (Findlay et al. 2011). The increased tube growth rates in *Simplaria* sp. exposed to low pH seem to indicate a reallocation of energy away from long‐term survival toward calcification investment, which may be compounding the species risk to OA. This finding also highlights the need to measure traits linked to Darwinian fitness, as only measuring size or growth rates can misrepresent actual evolutionary responses to OA.

### Concluding remarks and applied relevance

The reciprocal transplant experiment we carried out provided evidence against the idea that either local adaptation or phenotypic plasticity are evolutionary strategies supporting increased fitness levels in populations of *Simplaria* sp. from the low‐pH habitat during an abnormally intense venting period at the Castello CO_2_ vents. These results indicate that actual adaptive constraints to low pH as a selective driver can exist for this and other calcifying species and/or populations, which may be particularly relevant when the intensity and duration of pH exposure surpasses historically known variation for such populations (Parker et al. 2010; Kelly et al. 2013). This idea aligns well with the general notion that near‐future low‐pH projections will act as a severe threat particularly to marine calcifiers in highly variable regions such as coastal areas, lowering their ability to persist through the next century (Christen et al. 2013; Dupont and Pörtner 2013).

The temporal scale of OA may also compound these concerns. OA may exert a selective force on current marine populations that is too strong for adaptation within the given time frame (Reznick and Ghalambor 2001; Carroll et al. 2007), despite evidence that certain marine calcifying species are able to rapidly adapt to climate changes (Schluter et al. 2014). Ocean acidification is occurring at a rate ten times faster than any time in the last 55 million years (Hartmann et al. 2013). The pH range in some areas of the Castello CO_2_ vent site is similar to that predicted in global oceans by 2100 if high CO_2_ emissions continue (RCP 8.5, 50–100 years), but the vents have likely been active for approximately 2000 years (Lombardi et al. 2011a). This time lag may have provided local species and populations with a considerably longer time period to colonize and adapt than future marine populations will have. The lack of adaptive evolutionary responses in the benthic *Simplaria* sp. within the tested populations at this site, however, alludes to the possibility that the rate and magnitude of future OA conditions may not bode well for the maintenance of high levels of biodiversity in all marine species through evolutionary responses. Subsequently, we may expect an increase in extinction rates of certain calcifying species under future OA conditions, as already documented through the geological record (Benton and Twitchett 2003; Veron 2008). This would likely have important consequences for marine ecosystem functioning (Solan et al. 2004).

This study demonstrates the functionality of using natural pH gradients with F2 generation recruits in an *in situ* reciprocal transplant experiment to test for the presence of local adaptation. This experimental approach is powerful in that it relies on the natural evolutionary pathways that populations have previously experienced – pathways that are impossible to exactly replicate in breeding experiments or experimental evolution (Bell and Collins 2008). Furthermore, this approach is considered to be the best way to assess whether changes are adaptive by separating environmental effects from genetic effects (Nuismer and Gandon 2008; Merilä and Hendry 2014), and as such can directly help us determine the importance of plasticity as a mechanism enabling adaptive evolution (Munday et al. 2013; Sunday et al. 2013). Predictably, the diversity of phenotypic plasticity responses (i.e. nonadaptive or adaptive plasticity) and the time scales in which they are presented [i.e. intergenerational or transgenerational plasticity (TGP)] make it difficult to determine the role plasticity will play in evolutionary change (West‐Eberhard 2003; Ghalambor et al. 2007; Chakravarti et al. 2016; Rodríguez‐Romero et al. 2015). Expanding the basic reciprocal transplant approach to include common garden experiments performed under both low and ambient pH ‘common’ conditions, would help to broaden our understanding of plasticity as a mechanism of rapid adaptation by relating adaptation to TGP (Parker et al. 2015; Rodríguez‐Romero et al. 2015; Thor and Dupont 2015; Chakravarti et al. 2016; Ross et al. 2016).

In this study, neither local adaptation nor plasticity were found to improve the fitness of a calcifying tubeworm population from low‐pH vents, yet our results illustrate how realistic natural *in situ* plastic and adaptive responses can be multifaceted. We suggest that the *in situ* reciprocal transplant approach be used as a means to improve our limited knowledge of contemporary evolution of marine species under global change. The approach can also be used as a tool to refine future research with aims that can produce coordinated evolutionary predictions within the context of global change. Improving our understanding of how and why natural populations succeed or fail to adapt to OA, and global change drivers in general, will help guide resource management and conservation efforts (Palumbi 2001; Ashley et al. 2003; Stockwell et al. 2003; van Oppen et al. 2015).

## Data archiving statement

Data for this study are available at PANGAEA: https://doi.pangaea.de/10.1594/PANGAEA.861355.
